# Effect of COVID-19 Pandemic Lockdowns on Body Mass Index of Primary School Children from Different Socioeconomic Backgrounds

**DOI:** 10.1186/s40798-024-00687-8

**Published:** 2024-03-01

**Authors:** Ludwig Piesch, Robert Stojan, Jochen Zinner, Dirk Büsch, Katharina Utesch, Till Utesch

**Affiliations:** 1 Institute of Educational Sciences, University of Münster, Bispinghof 5/6, 48143 Münster, Germany; 2 Deutsche Hochschule für Gesundheit und Sport, Berlin, Germany; 3 Universität Oldenburg, Oldenburg, Germany; 4 Institute of Psychology, University of Münster, Münster, Germany; 5 Institute of Human Movement Science, University of Hamburg, Hamburg, Germany

**Keywords:** Childhood overweight, Childhood obesity, Physical literacy, BMI z-score, BMI standard deviation score

## Abstract

**Background:**

Childhood obesity is associated with various health outcomes. Restrictive measures to contain the spread of the Coronavirus Disease 2019 (COVID-19) pandemic, like lockdowns and school closures, affected children’s daily structure, physical activity, dietary habits, and sleep quality, possibly exacerbating risk factors for childhood obesity and higher body mass index (BMI) in children. Poor socioeconomic conditions may have led to relatively higher risk for elevated BMI levels following pandemic measures. In this study, the impact of measures related to the COVID-19 pandemic on the BMI of third graders was investigated regarding children’s socioeconomic background (SEB).

**Methods:**

Data from 41,728 children (8.84 ± 0.56 years, 20,431 female) were collected in the context of a cohort study. Children were tested either before the pandemic (pre_COVID_: Sept2017–March2020, *n* = 26,314), or following the first (post_LDI_: Aug2020–Dec2020, *n* = 6657) or second lockdown in Germany (post_LDII_: Aug2021–Jan2022, *n* = 8757). SEB was based on the official school type classification of the state of Berlin. Outcome was BMI standard deviation scores (SDS).

**Results:**

Significant effects of Time and SEB revealed elevated BMIs in post_LDI_ (*M* = 0.23, *p* = 0.011) and post_LDII_ (*M* = 0.22, *p* = 0.011) compared to pre_COVID_ (*M* = 0.17) cohorts and higher BMIs for children with lower SEB (*b* = − 0.13*, p* < 0.001). A significant Time × SEB interaction indicated that the effect of SEB on children’s BMI increased in response to lockdowns, especially in post_LDII_ (*b* = − 0.05*, p* = 0.006). Results suggest that the COVID-19-related measures lead to increased BMI in children, and that children of lower SEB were at particular risk for higher BMIs following lockdowns.

**Conclusions:**

These findings highlight the dependency of children’s BMI on societal circumstances. Over the course of two lockdowns in Germany, children have experienced BMI increments, particularly in low socioeconomic areas. Authorities are called into action to counteract increasing rates of childhood weight by promoting physical activity of children and establishing related post-pandemic offers.

**Supplementary Information:**

The online version contains supplementary material available at 10.1186/s40798-024-00687-8.

## Key Points

1. Children’s weight status and health could be affected in the short and long term as a consequence of increased BMI in primary school cohorts following COVID-19 pandemic-related lockdowns.
2. Children from lower socioeconomic backgrounds are at particularly high risk of poor weight status and have higher BMIs. This socioeconomic gradient increased in post-lockdown cohorts, which is why the health of socioeconomically disadvantaged children may have been disproportionally affected by the pandemic.
3. Authorities and policymakers are called upon to take countermeasures to minimize the impact of the COVID-19 pandemic on children’s weight status and to counteract risks of increasing childhood obesity rates, especially in socioeconomically poorer regions.

**Supplementary Information:**

The online version contains supplementary material available at 10.1186/s40798-024-00687-8.

## Background

Childhood obesity is a major public health challenge of the twenty-first century, and its worldwide prevalence has dramatically increased over the past four decades [1, 2]. Elevated body mass index (BMI) and obesity during childhood carry substantial immediate and long-term consequences, including increased risk of developing noncommunicable diseases (NCDs) such as cardiovascular diseases and diabetes mellitus as well as impaired psychosocial health [3–6]. Moreover, being overweight or obese in childhood increases the likelihood of lifetime overweight and obesity [7–10]. Therefore, combating childhood overweight and obesity has become a prioritized concern for public health authorities [11]. With the outbreak of the Coronavirus Disease 2019 (COVID-19) pandemic, however, recent efforts to counteract worldwide obesity trends and associated consequences may have been undermined.

With the aim of containing the spread of COVID-19, governments across the globe implemented an unprecedented range of policies and (non-pharmaceutical) measures since the beginning of 2020, including travel restrictions, bans on public gatherings, school and sport club closings, or even nationwide lockdowns with stay-at-home orders [12, 13]. While being necessary and effective in controlling the spread of the virus, these measures abruptly changed various daily habits of people. With schools being closed, especially children had limited opportunities to interact with their peers or to engage in physical activities for extended periods, depriving them of age-adequate cognitive and physical stimulation [14, 15]. In fact, findings from the pre-COVID-19 era suggest that when disengaged from their usual school curriculum (e.g., during summer recess, holidays), children tend to be less physically active, increase their screen-time, have irregular sleep schedules, and display unhealthier dietary behavior, leading to accelerated BMI gains and increased body fat [16–19]. Thus, it is conceivable that such obesogenic behaviors are reinforced when children are confined to their domestic environments and deprived of outdoor activities and interaction with peers [20]. Recent evidence supports this assumption, indicating that during COVID-19 pandemic-related lockdowns, children consumed unhealthier foods and beverages, spent more time in front of screens, had deteriorated sleep quality, and were less physically active [21–25]. Accordingly, the COVID-19 pandemic and associated lockdowns may have exposed children to an increased risk of obesogenic behaviors and BMI gain, prompting an undesirable shift in their weight status and contributing to the pandemic of childhood obesity. Given the deleterious effects of excess weight gain during childhood, it is of utmost importance to identify whether changes in children's living conditions and behaviors under pandemic-related restrictions translated into a worsening of children's weight status.

### Socioeconomic Background, COVID-19, and Childhood Weight Status

Though pandemic restrictions applied to citizens irrespective of their socioeconomic background (SEB), children from lower SEBs may have been at particular risk for aggravated weight status following these measures. SEB disparities with respect to childhood weight status, overweight, and obesity levels are well-documented, and the prevalence of overweight and obesity in childhood appears to be closely related to children’s SEB, suggesting the existence of a socioeconomic gradient for childhood overweight and obesity [26]. According to a growing body of literature, the relationship between the socioeconomic environment of a child and overweight and obesity prevalence is geo-specific, demonstrating an inverse relation in industrialized, high-income countries, like Germany [27]. In these countries, the prevalence of childhood overweight and obesity is disproportionately higher in areas where socioeconomic conditions are low [2, 28, 29]. This imbalance is owed to the fact that the socioeconomic environment in which a child grows up exerts substantial influence on the presence and effect of risk factors related to the development and persistence of childhood overweight and obesity [28]. For example, several authors report poorer nutrition, lower physical activity levels, higher screen time, and higher sedentary time to be more common among children of lower SEBs, putting them at a relatively higher risk of elevated weight and BMI as compared to children of higher SEBs [26, 28–30]. Accordingly, it is assumed that socioeconomic inequalities manifest as inequalities in the prevalence and effect of risk factors for childhood overweight and obesity, and are reflected in imbalances in children’s weight status along the range of the SEB continuum.

While already at risk for increased BMI and unhealthier weight status by virtue of their SEB, children from lower SEBs may have been further disadvantaged by the COVID-19 pandemic. Given that measures like lockdowns or home confinement were imposed in the context of preexisting socioeconomic inequalities, it seems plausible that the impact of such measures varied according to SEBs and, eventually, augmented socioeconomic inequalities in child health [31, 32].

**Fig. 1:**
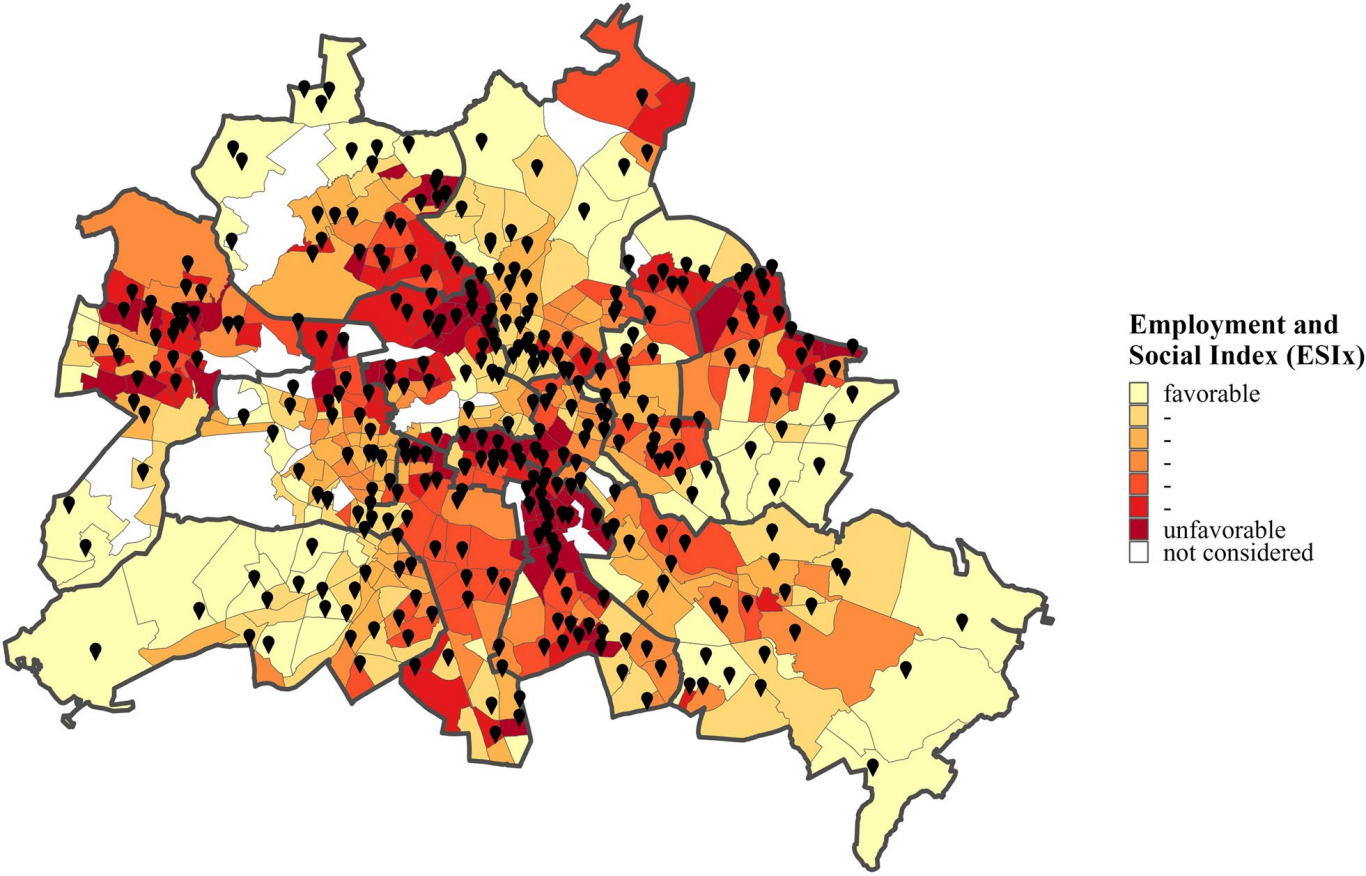
Location of schools which participated in the ‘Berlin has Talent’ program within the socioeconomically diverse city of Berlin. Schools are displayed as geo tag icons in black. Planning areas of Berlin are color graded according to the Employment and Social Index (ESIx) of the city of Berlin. The ESIx is adapted from the ‘Health and Social Structure Atlas of Berlin’ and informs about the economic and social wealth of a region. For more information about the ESIx, see Berlin Senate [37]

In fact, some studies have demonstrated socioeconomic inequalities in the impact of restrictive measures on health- and weight-related behaviors of children [32–36]. According to these studies, behaviors that are considered protective against weight gain during childhood (e.g., physical activity, balanced diet, low screen time) declined relatively more during COVID-19 home confinement in children from families with lower socioeconomic characteristics (e.g., lower parental education, smaller dwelling type, poorer housing conditions). However, few studies have directly investigated the impact of childhood SEB on child weight status in the context of the COVID-19 pandemic. Therefore, examinations of understanding how the COVID-19 pandemic and associated lockdowns affected the relationship between children’s SEB and their weight status are needed. This is of particular relevance for regions and cities with considerable socioeconomic disparities within the population. Therefore, it was the aim of this study to examine the effects of the COVID-19 pandemic and associated lockdowns on the weight status of third grade primary school children from the state of Berlin (Germany), while specifically considering how the relation between children’s SEB and their weight status was affected in the context of the COVID-19 pandemic (Fig. 1).

## Methods

### Sample and Study Design

**Fig. 2:**
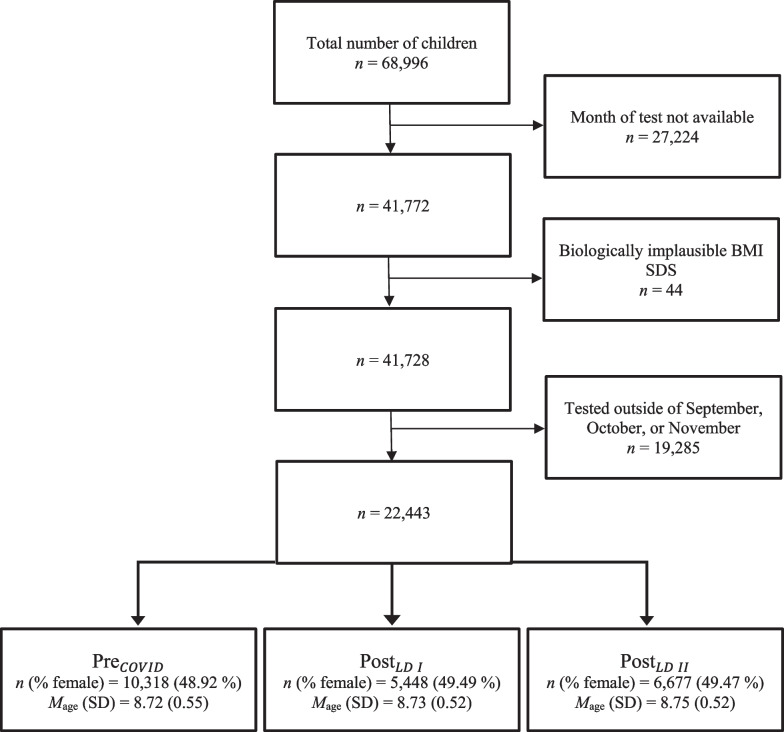
Flow diagram of excluded cases as well as demographic characteristics of the final sample by pandemic stage

**Fig. 3:**
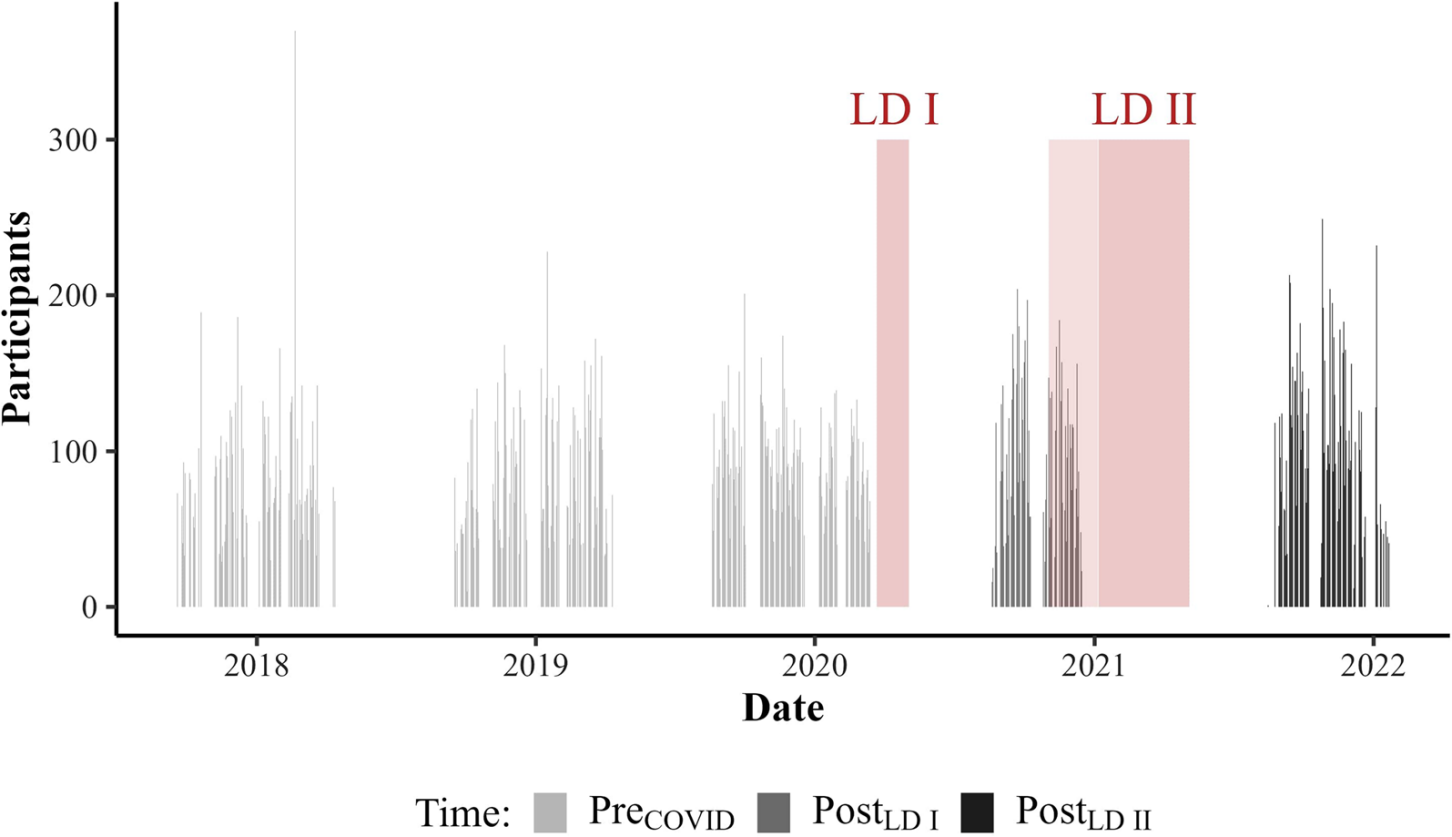
Data collection periods between September 2017 and January 2022. Measurements are displayed in grey by Time. Lockdowns are highlighted as red rectangles, the “lockdown light” (November 2020 until January 2021) is highlighted in light red. For more information on regulatory measures in Germany, see German Federal Statistical Office [Statistisches Bundesamt] [59].

The data analyzed in this cohort study were collected as part of the “Berlin has Talent” program (https://berlin-hat-talent.de/), a project of the Regional Sports Confederation Berlin [Landessportbund Berlin, LSB] and the Berlin Senate concerned with monitoring and promoting motor performance of third-grade primary school children in Berlin. The program, design, and recruitment process of the study have been extensively explained elsewhere [38–41]. The total data set comprised data from 68,996 children (8.83 ± 0.56 years, 33,726 female), of which 27,224 were excluded due to the unavailability of test dates [19, 42–44] and 44 due to biologically implausible BMI standard deviation scores (SDS) (BMI SDS > 8 & BMI SDS < -4) [45, 46]. To control for a potential seasonality bias, only data collected during the months September, October, and November were considered. This led to the exclusion of another 19,285 data points. The final sample for the analysis comprised data from 22,443 children (32.53% of those tested; 8.73 ± 0.53 years, 11,047 female) from 5 different cohorts. A flow diagram showing the number of data cases excluded at each stage as well as demographic characteristics of participants by pandemic stage are presented in Fig. 2. The study design and data collection periods in each cohort relative to COVID-19 pandemic lockdowns in Germany are visualized in Fig. 3. The study and the consent forms signed by the subjects’ parents were approved by the Berlin Senate. The study was conducted in accordance with the Declaration of Helsinki.

### Procedure: Data Collection

Data collection in the present sample was carried out annually between August/September (depending on the start of the school year) and April. Data collection was terminated early in March and December 2020, respectively, due to the imposition of COVID-19 pandemic-related lockdowns in these months. Most recent data from school year 2021/2022 was available until January 2022 at the time this study was conducted.

Each year, public primary schools from annually increasing numbers of Berlin districts (2017/2018: 6/12 districts; 2018/2019: 7/12 districts; 2019/2020: 8/12 districts; since 2020/2021: 12/12 districts) received a written invitation by the Berlin school administration to participate in the project. In the present sample, between 106 and 177 primary schools participated each year. The selection of the third graders to be examined was carried out school by school by a project group commissioned by the LSB and the Berlin school administration. Between 6607 and 10,482 third graders per year completed the testing procedure in our sample, provided that parental consent was given. Entrusted by the Berlin school administration, a company specialized in the collection of sports data was responsible for conducting the German Motor Test and corresponding anthropometric measures (e.g., height, weight), using virtually unchanged personnel over the years. Demographic variables (e.g., age, gender) were collected via questionnaires. The study and the consent forms signed by the subjects were approved by the Senate of Berlin.

### Variables

*BMI and BMI SDS*. To assess lockdown- and SEB-related effects as well as their interaction effects on child weight status, BMI was used as dependent variable. To calculate BMI, height and weight were measured according to standardized procedures using a measuring tape attached vertically to a wall and a scale, respectively. Values were recorded to the nearest 1 cm and 100 g, respectively. BMI was calculated by dividing weight by the square of height [BMI = weight (kg)/height (m^2^)]. To account for age and sex-specific BMI variation in growing children, BMI raw scores were converted to sex-standardized BMI-for-age SDS (BMI z-scores) using the national BMI-for-age reference standard of Germany by Kromeyer-Hauschild et al. [47]. The 10th, 50th and 90th BMI percentile curves of the present sample relative to the Kromeyer-Hauschild et al. [47] population are presented in the supplementary material (Additional file 1: Fig. S2).

*SEB*. SEB was an independent variable. It was based on the scientifically-based official school type classification of the state of Berlin and was not influenced by any of the authors of this paper. It comprises a multidimensional index describing the structural and social conditions of a school. The index is measured by authorities, used for political decisions, and considered to be valid [48]. The Berlin state school type classification takes six characteristics into account: (1) The number of pupils in a school whose parents draw social welfare, (2) the number of pupils in a school whose common language at home is not German, (3) the number of pupils in a school with special educational needs, (4) the number of pupils in a school who repeat a grade, (5) the total number of teachers in a school relative to the required number of teachers according to the school’s schedule, and, (6) the status-index of the Berlin Senate Administration for Urban Development and Housing, a spatial index based on child poverty, unemployment benefits, and transfer payments. For index formation, each characteristic is divided into five equally sized segments, depending on their respective ranges, and, thus, equally weighted. A loading value (1–5) is assigned to each of the five segments, and the average of all loading values of a school gives its index. Based on this index, a school is classified as one of seven school types. The Berlin state school type classification is school-specific. Hence, the SEB value of a child represents the child’s SEB at the school-level.

*Time*. Time was an independent variable and defined with respect to government-imposed lockdowns during the COVID-19 pandemic in Germany. By the time this study was conducted, Germany experienced two major lockdowns, categorized as lockdown I (LD I: March 2020–May 2020, i.e., about three month) and lockdown II (LD II: January 2021–May 2021, i.e., about five month). Measurements prior to the 1st lockdown were operationalized as pre_COVID_ (September 2017–March 2020), following the 1st and prior to the 2nd lockdown as post_LDI_ (August 2020–December 2020), and following the 2nd lockdown as post_LDII_ (August 2021–January 2022; Fig. 3).

### Data Analysis and Statistics

All statistical analyses were performed with RStudio (Version 4.1.2) [49]. Pre- and post-processing of data were carried out using the *tidyverse* package [50]. Statistical inference regarding the effects of COVID-19 pandemic lockdowns and SEB on BMI of primary school children was based on multilevel mixed-effects models (*lme4* and *lmerTest* packages) [51, 52]. The outcome measure was age- and sex-adjusted BMI SDS (continuous, higher values indicate higher BMI). BMI raw scores were converted to BMI SDS using the *sds* function of the *childsds* package [53]. Independent variables were Time (pre_COVID_, post_LDI_, post_LDII_), school-level SEB (continuous, − 2 to 2, higher values indicate higher SEB), and the interaction thereof, Time × SEB. The two lowest and highest of the seven SEB groups were aggregated to one group, respectively, due to low incidence in the lowest and highest SEB group, respectively. Covariates were Age (continuous, z-standardized), Gender (male, female) [54], and Month of Test (Jan., Feb., … Nov., Dec.). Month of Test was added to control for seasonal variation of BMI (e.g., school holidays, seasons [19, 42–44]) in addition to excluding data which were not collected in September, October, or November from the analysis. The supplement includes results of the analysis when all available test months were used (Additional file 1: Tables S8–S14). Independent variables were added as fixed effects as well as covariates to control for potential confounding effects. A random effects term was specified for School and District, with participants (level 1) being nested in School (level 2, intraclass correlation coefficient_school_ [ICC_school_] = 0.05) and District (level 2, ICC_district_ = 0.02). This allowed us to account for the variance arising from differences between School and District and adjust the model estimates accordingly. We adhered to a bottom-up model building approach, starting with a baseline model and evaluating whether the inclusion of additional components adds to the fit of the model [55, 56]. Likelihood ratio tests were applied to evaluate fixed effects via improved model fit for less parsimonious models (*anova* function; *R base* package). Model testing started by evaluating the random intercept-only model (unconditional means model, M0) and the covariate model (M1), containing the control variables Age, Gender, and Month of Test, and the nested random intercept structure. Subsequently, Time (M2), SEB (M3), and Time × SEB (M4) were successively added to evaluate main and interactions effects of independent variables using $\chi^{2}$ likelihood ratio tests. Extending the random effects structure (i.e., by adding random slopes for the independent variables) led to convergence and singularity issues. According to parsimonious model fitting principles [51], only random intercepts for School and District were included in the final model. Contrast analyses were performed based on the final model using estimated marginal means (*emmeans* package) [57] to contrast pre_COVID_, post_LDI_ and post_LDII_ BMI SDS. Estimated marginal trends (*emtrends* function; *emmeans* package) [57] were used to inspect the Time × SEB interaction and investigate to which extent the effect of SEB (i.e., slope) on BMI SDS varied as a function of Time, that is, between pre_COVID_, post_LDI_ and post_LDII_. Unstandardised ($b$) and standardised ($\beta$) regression weights as well as 95% confidence intervals of these comparisons are reported (Additional file 1: Tables S5 and S7), and *p*-values were Benjamini–Hochberg adjusted to control for false discovery rate, that is, type-I-error [58]. All models were fitted using maximum likelihood estimation (ML), and all tests were performed at a significance level of α = 0.05 to control for type-I error. *F*-statistics were computed to facilitate interpretation of main and interaction effects using type-III sum of squares ANCOVA with Satterthwaite-approximation (*anova* function; *R base* package; Additional file 1: Table S1).

## Results

3.1 OverviewThe distribution of the data by Time and SEB is displayed in the supplement (Additional file 1: Fig. S3). Likelihood ratio tests of stepwise model comparisons are presented in Additional file 1: Table S2. According to the unconditional means model (M0), there was substantial BMI SDS variance between School (τ_00_ = 0.04, *SD* = 0.21, 95% CI 0.18, 0.23) and District (τ_00_ = 0.02, *SD* = 0.16, 95% CI 0.09, 0.28) as well as within them (σ^2^ = 1.19, *SD* = 1.09, 95% CI 1.08, 1.1), providing evidence for the mixed-effect model approach. The parameter estimates of the final model including fixed effects of covariates, predictors, and interaction terms alongside random effects for School are presented in Additional file 1: Table S3. Unstandardized and standardized regression values are given with their confidence intervals and *p*-values for each predictor. Details on the estimated marginal means and estimated marginal trends analysis are presented in Additional file 1: Tables S4–S7. *F*-statistics of the final model computed using a type-III sum of squares ANCOVA with Satterthwaite-approximation can be found in Additional file 1: Table S1.

### Lockdown-Related Effects

**Fig. 4:**
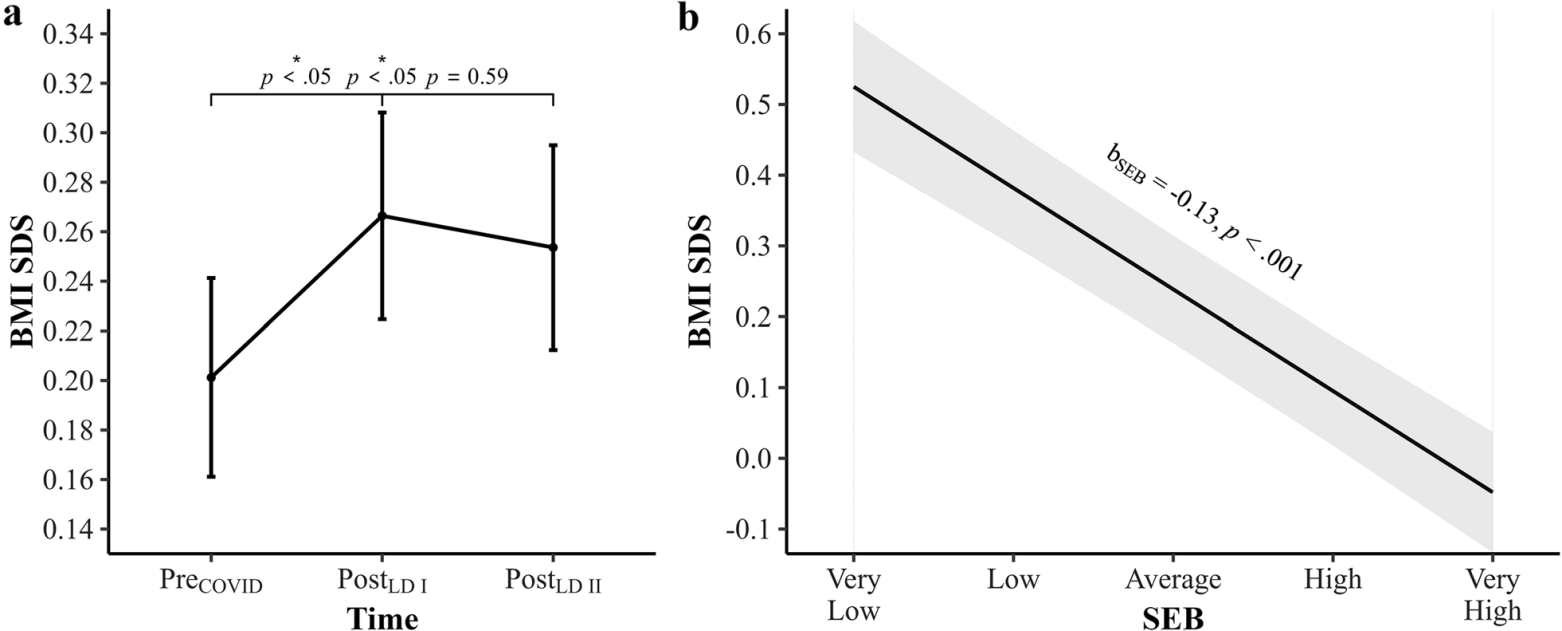
Main effects of Time and SEB on BMI SDS. **a** Post-hoc pairwise comparisons (contrasts) of estimated marginal mean BMI SDS with standard errors by Time (pre_COVID_, post_LDI_ and post_LDII_). Benjamini–Hochberg adjusted p-values from the estimated marginal mean analysis are given. **b** Effect of SEB on BMI SDS (Slope). Unstandardized beta values and p-values from multilevel model estimates are given

Likelihood ratio tests revealed that the main effect of Time, $\chi^{2}$ (2) = 9.74, *p* = 0.008, was significant (Additional file 1: Table S2), suggesting considerable differences in BMI SDS between stages of the COVID-19 pandemic. Post-hoc estimated marginal means analysis determined that BMI SDS in post_LDI_ (*M* = 0.27, *SE* = 0.04) and post_LDII_ (*M* = 0.25, *SE* = 0.04) were higher compared to BMI SDS in pre_COVID_ (*M* = 0.20, *SE* = 0.04; Additional file 1: Table S4). According to post-hoc pairwise comparisons (contrasts), the BMI SDS differences between pre_COVID_ and post_LDI_ (*p* = 0.012) and between pre_COVID_ and post_LDII_ (*p* = 0.024) were both significant, indicating that BMI levels in Berlin third grade primary school children were significantly elevated in response to the COVID-19 pandemic-related lockdowns. There was no significant difference between post_LDI_ and post_LDII_ BMI SDS levels (*p* = 0.588), suggesting elevated BMI SDS across all children in post_LDI_ persisted in post_LDII_ (Additional file 1: Table S5). Results of the estimated marginal means analysis and post-hoc pairwise comparisons between BMI SDS of each pandemic stage are illustrated in Fig. 4a.

### SEB-Related Effects

There was a significant effect of school-level SEB on BMI SDS, $\chi^{2}$ (1) = 109.71, *p* < 0.001, according to likelihood ratio tests (Additional file 1: Table S2), suggesting that BMI SDS varied substantially as a function of a child’s SEB. Estimates of the multilevel model indicate that BMI SDS of Berlin third grade primary school children was lower with higher SEB and was higher with lower SEB (*p* < 0.001). Thus, results are in support of a socioeconomic gradient for children’s weight status. The linear effect of school-level SEB on BMI SDS is portrayed in Fig. 4b.

### *Time* × *SEB Interaction Effects*

**Fig. 5:**
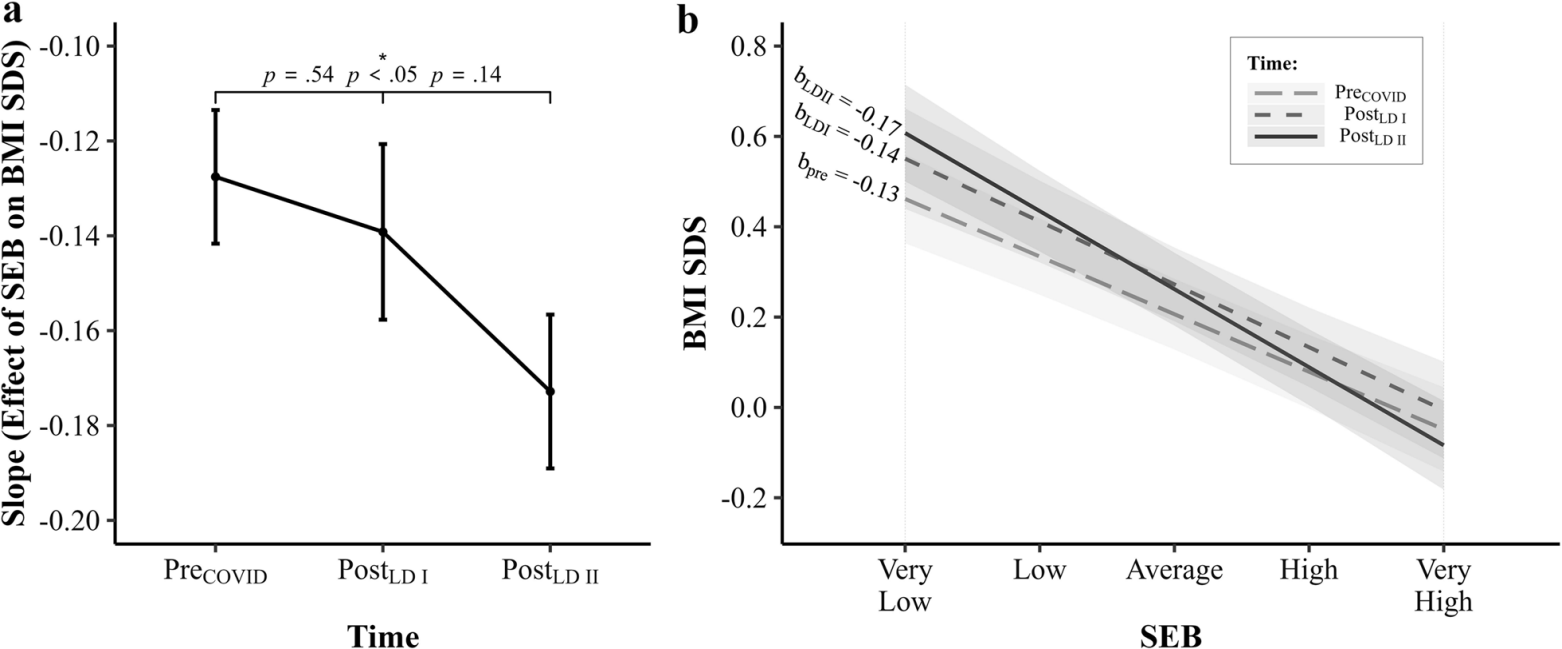
Interaction effect of Time × SEB on BMI SDS. **a** Post-hoc pairwise comparison of slopes (estimated marginal trends with standard errors) in pre_COVID_, post_LDI_ and post_LDII_. Benjamini–Hochberg adjusted p-values from estimated marginal trends analysis are given. **b** Effect of SEB on BMI SDS (slopes) in pre_COVID_, post_LDI_ and post_LDII_ according to estimated marginal trends analysis. Beta values (estimated marginal trends) are given

After adding the interaction term Time × SEB to the model, likelihood ratio tests disclosed a significant interaction effect, $\chi^{2}$ (2) = 6.92, *p* = 0.031 (Additional file 1: Table S2), describing that the effect of SEB on children’s BMI SDS varied significantly as a function of Time, that is, depended on the stage of the COVID-19 pandemic. Post-hoc estimated marginal trends analysis revealed that the effect of SEB on BMI SDS (i.e., the slope) slightly increased (that is, the slope exhibited a steeper decline) from pre_COVID_ (b_pre_ = − 0.13, *SE* = 0.01) to post_LDI_ (b_postLDI_ = − 0.14, *SE* = 0.02), and noticeably increased in post_LDII_ (b_postLDII_ = − 0.17, *SE* = 0.02; Fig. 5; Additional file 1: Table S6). Thus, the linear effect of SEB on BMI SDS was highest in the post_LDII_ stage, followed by the post_LDI_ and the pre_COVID_ stage. Post-hoc pairwise comparisons of SEB-slopes (slope contrasts) showed a significant difference between pre_COVID_ and post_LDII_ SEB-slopes (*p* = 0.028). However, the differences between post_LDI_ and post_LDII_ SEB-slopes (*p* = 0.142) and between pre_COVID_ and post_LDI_ SEB-slopes (*p* = 0.54, Fig. 5a) were not significant. As such, the linear effect of SEB on BMI SDS was not significantly stronger in post_LDI_ compared to pre_COVID_, but significantly elevated in post_LDII_ compared to pre_COVID_ (Fig. 5; Additional file 1: Table S7). In other words, the effect that children with lower SEB have higher BMIs just slightly increased in post_LDI_, but substantially increased in the post_LDII_ stage. Post-lockdown BMI trajectories by SEB (Additional file 1: Fig. S1) show that BMIs of children with higher SEB even declined from post_LDI_ to post_LDII_, while BMIs of lower SEB children exhibited an incline in this period.

## Discussion

The findings of this study indicate that (1) age- and sex-adapted BMI SDS were considerably elevated in children measured after the 1st and 2nd lockdown in Germany, (2) children with lower school-level SEB had substantially higher BMI SDS across all cohorts than those with higher school-level SEB, and (3) the socioeconomic gradient reflecting significantly higher BMI SDS for children with lower SEB was even stronger in post-lockdown cohorts, especially in the post-lockdown II cohort.

The results of our study coincide with what several authors predicted following the global outbreak of the Coronavirus, that is, weight gain in children in response to exacerbated obesogenic circumstances during lockdown-inflicted home confinement [20, 60–62]. During lockdowns of the COVID-19 pandemic, children were deprived of structured institutional educational environments, a setting which Brazendale et al. [16] describe to protect children against weight gain by regulating obesogenic behaviors (e.g., through compulsory and non-compulsory physical activity opportunities, restricting caloric intake, reducing screen time occasions, and regulating sleep schedules). With this protective environment being less accessible or even inaccessible and age-adequate cognitive and physical stimuli being deprived, Rundle et al. [20] proposed that obesogenic environments conducive to physical inactivity and poor diet are reinforced during COVID-19 home confinement, exposing children to an increased risk for energy imbalance and accelerated weight gain. These assumptions are supported by more recent findings from the field, indicating that children indeed made persisting unhealthy dietary and lifestyle behavioral changes during COVID-19 lockdowns [21–25]. The findings from our study, in turn, are among the first to point to the idea that this shift in dietary and lifestyle behaviors caused by the COVID-19 pandemic and associated lockdowns plausibly translates to weight and BMI gain in children, constituting a threat to the combat of childhood overweight and obesity. Recent studies comply with our findings and report increases in BMIs among children during the COVID-19 pandemic. For instance, Lange et al. [63] assessed BMI trends during the pandemic in a geographically diverse sample of over 400,000 U.S. children and adolescents and found that the rate of BMI increase nearly doubled in pandemic periods. Importantly, the largest increase occurred in those with pre-pandemic overweight and obesity and in an age group that is comparable with our sample (6–11-year-old). In another recent study of almost 200,000 racially and ethnically diverse U.S. children and adolescents, Woolford et al. [64] reported sizable BMI gain during the pandemic, which was highest among 5- to 11-year-olds. Comparable findings were made in Central Europe by Jarnig et al. [65] who observed BMI increases in a population of 764 7–10-year-old Austrian children between September 2019 and September 2020. Thus, together with our study, accumulating evidence suggests that measures to mitigate the spread of the COVID-19 pandemic like lockdowns have led to exacerbated weight status and BMIs in primary school-aged children. Given that elevated BMI during childhood is not only predictive for worsened lifetime weight status and related conditions [7–10], but also strongly negatively associated with childhood motor competence and physical activity [66, 67], there is reason to worry that the observed changes in weight status could persist and affect children at different levels for years to come. Recently published data suggests that various domains of motor performance may have already been compromised as a consequence of the pandemic [68, 69].

As regards SEB disparities in BMIs, our findings are consistent with previous insights pointing to an inverse relation between childhood SEB and weight status [2, 26], in particular in high-income countries like Germany [27]. In their review, Vazquez and Cubbin [28] identified several mechanisms that may explain SEB-related inequalities in childhood weight status and obesity prevalence. According to the authors, various factors such as lack of access to grocery stores with healthy food, lower cost per calorie of more energy dense foods, lack of safe space to engage in physical activity, and low interest or awareness of weight control are more prevalent in low socioeconomic environments, possibly accounting for higher BMIs and obesity prevalence in child populations with lower SEB. Thus, the socioeconomic gradient for childhood BMI that we observed in our data is in line with previous research.

What our study contributes to established research in this field is evidence of socioeconomic inequalities in how children’s weight status is affected by the COVID-19 pandemic. Our results suggest that already existing socioeconomic-related differences in children’s BMIs (i.e., higher BMIs in low SEB children and vice versa) were reinforced in response to lockdowns and that the preexisting socioeconomic gradient displayed an even stronger decline after government-imposed lockdowns, in particular after the 2nd lockdown (Fig. 5). The observed increase in the SEB effect from post_LDI_ to post_LDII_ appears natural given the duration of both lockdowns. With almost six months in duration, the 2nd lockdown endured substantially longer than the 1st lockdown (less than 2 months; Fig. 3) [59] and exposed children to the presumed obesogenic low SEB environments for a much longer period. However, it must be mentioned here that the 2nd lockdown, although longer, was less strict in terms of regulatory measures (for details on regulatory measures during the COVID-19 pandemic in Germany, see: German Federal Statistical Office [Statistisches Bundesamt]) [59]. Yet, based on the presented data, we cautiously infer that the duration of home-confining measures plays an eminent role with respect to their impact on the association between SEB and childhood BMI. Moreover, the data indicates that children with higher SEB were at least partially able to make up for the initial BMI gain from post_LDI_ to post_LDII_, while in contradiction to this, BMIs of those with lower SEBs notably inclined in this period (Additional file 1: Fig. S1). Thus, our findings suggest that the detrimental effects of home-confinement on weight status manifest particularly in lower SEB children and may further increase evident socioeconomic weight status disparities in children along the SEB dimension. These results are in line with Jenssen et al. [70] who explored changes in childhood obesity disparities in response to the COVID-19 pandemic based on age, race and ethnicity, insurance, and income in a large and diverse U.S. sample. The authors found that preexisting disparities in childhood obesity rates in terms of race and ethnicity, insurance, and neighborhood socioeconomic status expanded during the pandemic. Measures and efforts to mitigate the number of COVID-19 infections like lockdowns, thus, have likely widened the gap between children from low and high SEBs in terms of weight status disparities. We do not know the exact pathways through which the COVID-19-related lockdowns had an unequal impact on child weight status. However, some authors have suggested potential mechanisms that could be conducive to socially unequal BMI gain in children during the pandemic. Abrams and Szefler [71] argue that school closures during the pandemic could increase malnutrition and food insecurity in low SEB children, both of which are closely linked to childhood SEB and weight status [28], as they can no longer profit from school lunch programs. Moreover, the financial losses expected as a consequence of the pandemic could constrain the budget of low-income household for unprocessed whole foods [61], and missing out on (subsidized) food services provided by schools could impose additional food-related financial burdens [72]. Based on the lower cost per calorie of more energy dense foods (i.e., fast foods), the pandemic may therefore reinforce the preexisting propensity of low SEB families to purchase these foods [28, 62]. Furthermore, González-Rábago et al. [32] proposed that children from families with low educational and financial levels lived in poorer housing conditions during lockdowns with limited space for physical activity and sociability, possibly disproportionately affecting weight status of children in these families. Finally, based on the assumption that low SEB environments and households are more conducive to obesogenic behaviors in children than high SEB environments and households [26, 28, 29], being exposed to these environments for extended periods likely exacerbates weight status in children from lower SEBs, but less so in children from higher SEBs. Future studies should identify mechanisms and factors that explain pandemic-related increments in social inequalities of weight status and BMI in children.

In summary, the overall results point to an alarming trend in the developments of primary school-aged children’s weight status. The weight status of children could be sustainably compromised as a result of pandemic lockdowns. Notably, children from lower SEBs seem to be at particular risk of exacerbated weight status in response to lockdown periods, likely contributing to increasing social disparities in the prevalence of childhood overweight and obesity. Second, our findings raise concerns that the stabilization in BMI trends and childhood obesity prevalence, which was recently observed in Germany and other high-income countries [2, 27], could have been destabilized as a consequence of the COVID-19 pandemic. This trend undermines public health efforts to combat childhood obesity. Importantly, these effects could be magnified in countries where lockdown regulations and home confinement orders were even stricter than in Germany. Third, based on the long-term adverse health outcomes of elevated BMI levels in childhood, our findings imply the urgent necessity for policymakers and various stakeholders (e.g., schools, communities, and families) to design and implement countermeasures to minimize the detrimental impact of the COVID-19 pandemic on children’s weight status. Promoting physical activity and establishing post-pandemic offers such as tailored physical health and nutrition education or weight management care is warranted to counteract risks of increasing rates of childhood obesity and related conditions.

Our study is subject to some limitations. First, as we analyzed secondary data, the choice of covariates was restricted to the available measures. Several important covariates (e.g.,, sport club membership, physical activity, sedentary behavior, dietary behavior), which affect BMIs in children [7] and have shown to be influenced by the pandemic [21–25], were not examined but could provide additional insights into pandemic- and SEB-related effects on BMIs in children. This may also explain why overall explanatory power of our model is low (conditional R, Additional file 1: Table S3) despite significance of effects. Second, though cohorts in this study included a socioeconomically diverse sample, data restricted to the county Berlin may not be nationally representative. Therefore, this study should be replicated with population-based data. Third, BMI is criticized for being limited in differentiating body fat from lean (fat free) mass. However, it is the most widely applied criterion of weight status and an accurate anthropometric indicator of body fat in 7-to-10-year-olds [73], especially when adjusted to age and sex-specific standards. Moreover, BMI is easy and safe to obtain in large child populations and a well-tolerated alternative to invasive measurement methods of weight status and body fat [74]. Fourth, we could not compare our cohorts against a control group of children unaffected by COVID-19 lockdowns. Therefore, causal inference with respect to observed effects cannot be made. Fifth, we cannot rule out the possibility of cohort effects, even though between-cohort variance in BMI SDS was negligible (ICC = 0.0001).

## Conclusion

In this cohort study of German third grade primary school children, considerable increases in BMIs were observed in response to lockdowns and home confinement during the COVID-19 pandemic. BMI increases following lockdowns were comparatively largest in children with low SEB, raising concerns that social disparities with respect to weight status and obesity prevalence in children may be widening as a consequence of the pandemic. These observations complement recent results on the development of BMI and weight status trends in times of the COVID-19 pandemic [63–65]. Our findings underscore the urgent need of countermeasures and post-pandemic efforts to mitigate increasing risks of childhood obesity prevalence and oppose adverse long-terms effects on child health. This is relevant not only in the context of the COVID-19 pandemic, but also with respect to future pandemics or crises that could promote changes in obesity-related lifestyles and behaviors (e.g., climate change-related altering of physical activity behavior). Providing weight management care to children, for example, in form of tailored physical health and nutrition education programs, is a critical task for states, communities, and schools to alleviate collateral damage of the COVID-19 pandemic. Finally, our findings should help to inform future pandemic policies.

### Supplementary Information

**Additional file 1.** Supplementary tables and figures for the analysis and result tables of the data analysis across all available test months.

## Data Availability

The datasets analyzed in this study can be requested from Till Utesch. The R code  used to process and analyze the data is available in the Open Science Framework (OSF) Repository https://osf.io/bc4uj/.

## Abbreviations

BMI: Body mass index
COVID-19: Coronavirus disease 2019
NCDs: Noncommunicable diseases
SDS: BMI standard deviation scores
SEB: Socioeconomic background
