# Supplementary material for: Effect of COVID-19 Pandemic Lockdowns on Body Mass Index of Primary School Children from Different Socioeconomic Backgrounds
Source: Sports Med Open. 2024 Mar 1;10:20. doi: 10.1186/s40798-024-00687-8 (PMC10907325; doi:10.1186/s40798-024-00687-8)
Supplement: Supplementary file 1 — Additional file 1. Supplementary tables and figures for the analysis and result tables of the data analysis across all available test months. [file 40798_2024_687_MOESM1_ESM.docx]

*Journal*: Sports Medicine – Open

Effect of COVID-19 pandemic lockdowns on body mass index of primary school children from different socioeconomic backgrounds

Piesch, Ludwig^1,5^, Stojan, Robert^1^, Zinner, Jochen^2^, Büsch, Dirk^3^, Utesch, Katharina^4^ & Utesch, Till^1*^

Affiliations:

1 Institute of Educational Sciences, University of Münster, Münster, Germany

2 Deutsche Hochschule für Gesundheit und Sport, Berlin, Germany

3 Universität Oldenburg, Oldenburg, Germany

4 Institute of Psychology, University of Münster, Münster, Germany

5 Institute of Human Movement Science, University of Hamburg, Hamburg, Germany

## Supplement

**Details** **on model fitting**

### Based on a model with random intercepts for School and District and covariates (M1), successively adding the predictors Time (M2), SEB (M3) and the interaction thereof (M4) significantly contributed to goodness-of-fit, respectively (Table S2, supplement). Adding random slopes for the for Time by School and random slopes for SEB by District improved model fit but caused a linear dependency between random effects (i.e., singularity). In line with Bates et al. [51], a parsimonious model was fitted. Accordingly, random intercepts for School and District and all covariates, predictor variables, and variance components were included in the final model (M4).

### Figures


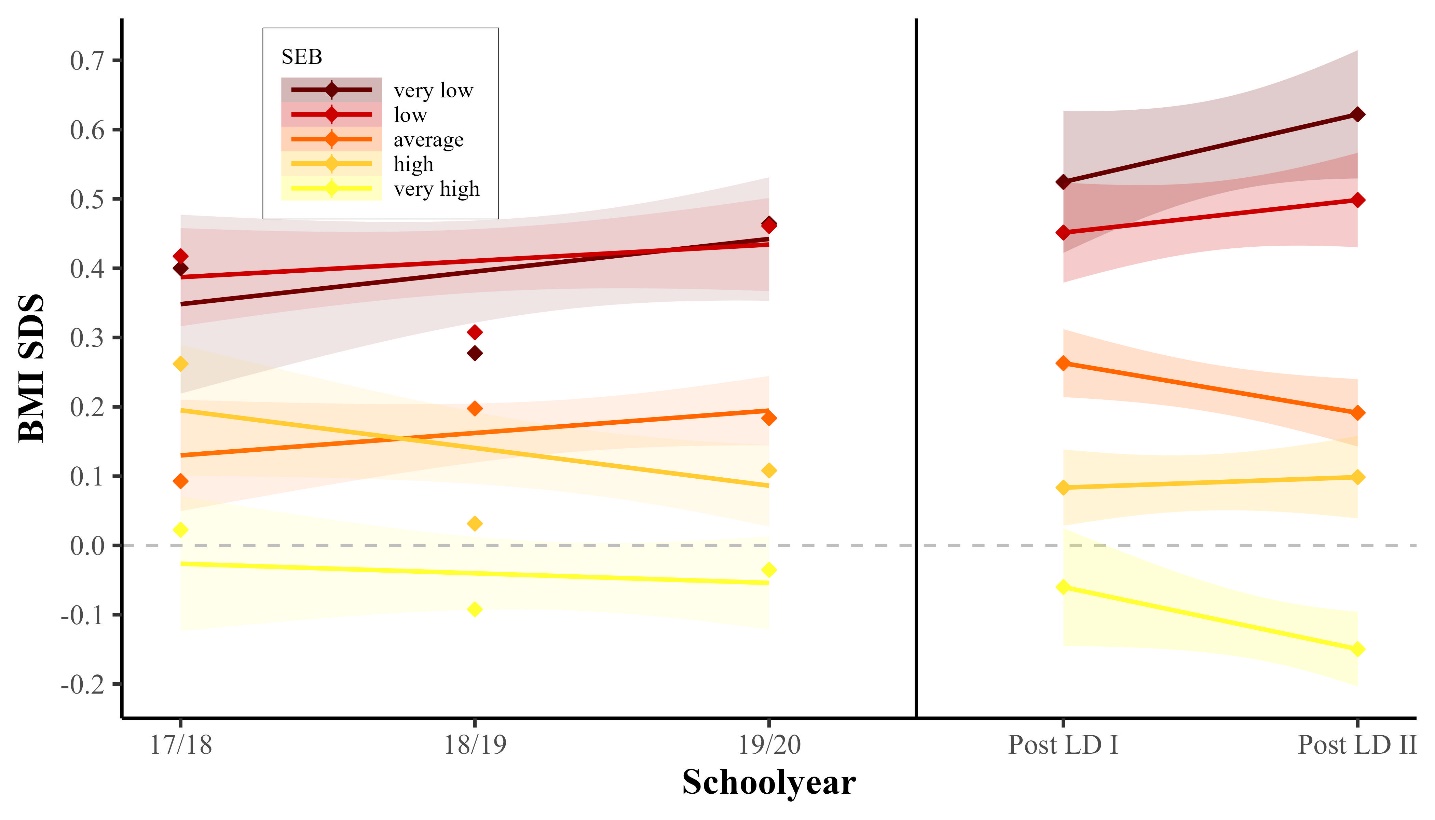


Figure S1. Secular trend of BMI SDS prior to and following the outbreak of COVID-19 pandemic by SEB. Linear trends and means for each schoolyear are presented.


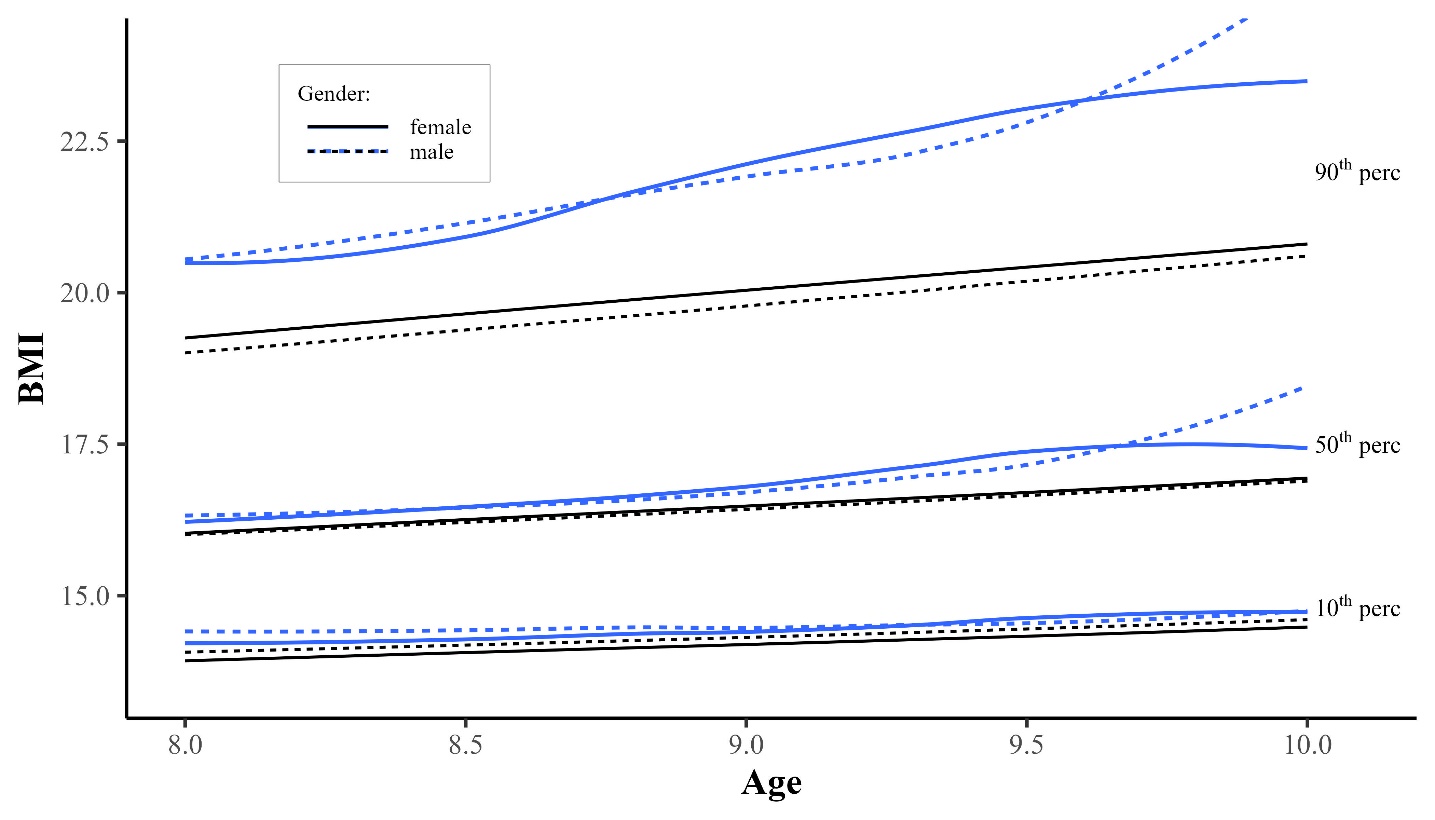


Figure S2. BMI percentile curves at 10${}^{th}$, 50${}^{th}$ and 90${}^{th}$ percentile for boys and girls aged 8-10. Percentile curves of the present sample are presented in blue and were smoothed using LOESS approximation. Percentile curves of the reference population by Kromeyer-Hauschild et al. (2001) are presented in black.


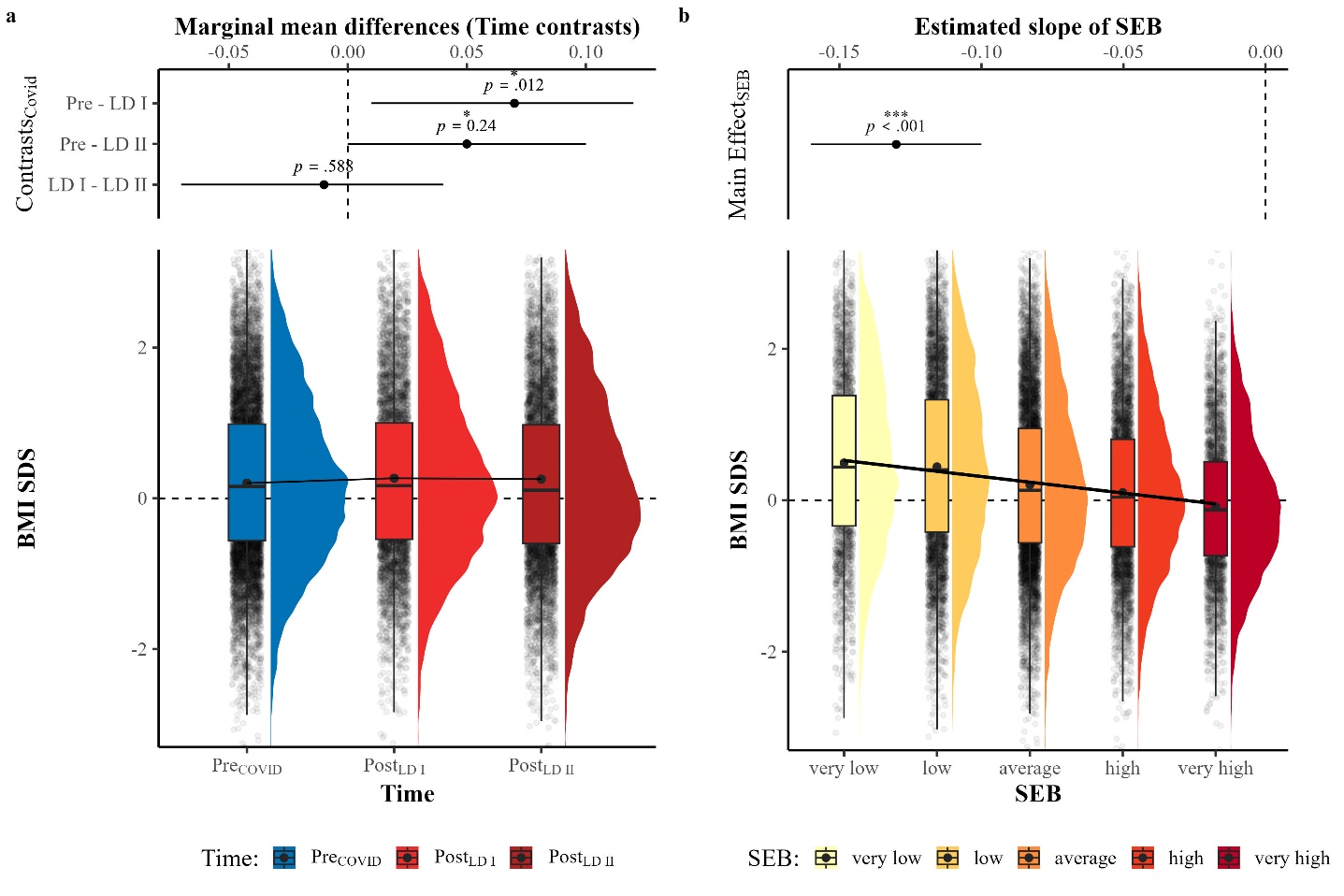


Figure S3. a) Effect of Time on BMI SDS. Top: Estimated marginal mean differences of Time BMI SDS contratsts. Bars are 95% confidence intervals of the effects. Benjamini-Hochberg adjusted p-values from estimated marginal means analysis are given. Bottom: Data distribution with mean and median by pandemic stage. Means are estimated marginal means determined by post-hoc analysis. b) Effect of SEB on BMI. Top: Estimated slope of SEB. Bars are 95% confidence intervals of the effect. Unadjusted p-value of the estimate is given. Bottom: Data distribution with mean and median by SEB group and the linear effect (slope) of SEB on BMI as estimated by the multilevel model.

### Tables

Table S1. Results of the type-III sum of squares ANCOVA table with Satterthwaite-approximation.

| **Predictor** | **Sum Sq** | **Mean Sq** | **DF** | **DenDF** | **F-value** | **Pr(>F)** |
| --- | --- | --- | --- | --- | --- | --- |
| Time | 12.57 | 6.29 | 2 | 2873.66 | 5.29 | **< .01** |
| SEB | 170.43 | 170.43 | 1 | 211.65 | 143.49 | **< .001** |
| Gender | 1.11 | 1.11 | 1 | 21948.43 | 0.94 | .33 |
| Age | 1.22 | 1.22 | 1 | 21944.28 | 1.03 | .31 |
| Month of Test | 6.56 | 3.28 | 2 | 1865.19 | 2.76 | .06 |
| Time x SEB | 8.30 | 4.15 | 2 | 2759.77 | 3.49 | **< .05** |

Table S2. Likelihood ratio tests of fixed effects.

| **Model** | **AIC** | **BIC** | **logLik** | **Chisq** | **Param** | ***p*** |
| --- | --- | --- | --- | --- | --- | --- |
| M0 vs. M1 |  |  |  |  |  |  |
| M0 | 67891.24 | 67923.31 | -33941.62 |  | (1\|School) |  |
| M1 | 67891.60 | 67955.75 | -33937.80 | 7.64 | Covariates | 0.106 |
| M1 vs. M2 |  |  |  |  |  |  |
| M1 | 67891.60 | 67955.75 | -33937.80 |  |  |  |
| M2 | 67885.85 | 67966.04 | -33932.93 | 9.74 | Time | **< .01** |
| M2 vs. M3 |  |  |  |  |  |  |
| M2 | 66588.10 | 66668.09 | -33284.05 |  |  |  |
| M3 | 66480.39 | 66568.38 | -33229.20 | 109.71 | SEB | **< .001** |
| M3 vs. M4 |  |  |  |  |  |  |
| M3 | 66480.39 | 66568.38 | -33229.20 |  |  |  |
| M4 | 66477.48 | 66581.47 | -33225.74 | 6.92 | Time x SEB | **< .05** |
| Note. Models were successively extended by the following parameters: M0: Unconditional means model with random intercepts for School. M1: Covariates Age, Gender, Month of Test. M2: Predictor Time. M3: Predictor SEB. M4: Predictor Time x SEB interaction. M5: Random slopes for Time. | | | | | | |

Table S3. Fixed and random effect estimates of the fitted (final) linear mixed effects model.

| **Predictor** | **Estimate** | **Std. Beta** | **CI** | **Std. CI** | ***p*** | **Std. *p*** |
| --- | --- | --- | --- | --- | --- | --- |
| Intercept | 0.17 | -0.05 | 0.09 – 0.26 | -0.13 – 0.02 | **<0.001** | 0.179 |
| Time[LD I] | 0.07 | 0.06 | 0.02 – 0.11 | 0.02 – 0.10 | **0.004** | **0.004** |
| Time[LD II] | 0.06 | 0.05 | 0.01 – 0.10 | 0.01 – 0.08 | **0.011** | **0.016** |
| SEB | -0.13 | -0.14 | -0.16 – -0.10 | -0.17 – -0.11 | **<0.001** | **<0.001** |
| Gender[m] | 0.01 | 0.01 | -0.01 – 0.04 | -0.01 – 0.04 | 0.333 | 0.333 |
| Age | 0.01 | 0.01 | -0.01 – 0.02 | -0.01 – 0.02 | 0.310 | 0.310 |
| Oct | 0.06 | 0.05 | 0.01 – 0.11 | 0.01 – 0.10 | **0.019** | **0.019** |
| Nov | 0.03 | 0.03 | -0.02 – 0.09 | -0.02 – 0.08 | 0.246 | 0.246 |
| Time[LD I] x SEB | -0.01 | -0.01 | -0.05 – 0.03 | -0.05 – 0.03 | 0.541 | 0.541 |
| Time[LD II] x SEB | -0.05 | -0.05 | -0.08 – -0.01 | -0.08 – -0.01 | **0.009** | **0.009** |
| **Random Effects** | | | | | | |
| σ^2^ | 1.19 | | | | | |
| τ_00_ _school_ | 0.02 | | | | | |
| τ_00_ _district_ | 0.01 | | | | | |
| ICC | 0.02 | | | | | |
| N _school_ | 226 | | | | | |
| N _district_ | 9 | | | | | |
| Observations | 22007 | | | | | |
| Marginal R^2^ / Conditional R^2^ | 0.024 / 0.048 | | | | | |

Table S4. Estimated marginal means (emmeans) and confidence intervals for BMI in each stage of the COVID-19 pandemic. Confidence level used: 0.95.

| **Time** | **emmean** | **SE** | **Lower CL** | **Upper CL** |
| --- | --- | --- | --- | --- |
| Pre_COVID_ | 0.20 | 0.04 | 0.12 | 0.28 |
| Post_LD I_ | 0.27 | 0.04 | 0.18 | 0.35 |
| Post_LD II_ | 0.25 | 0.04 | 0.17 | 0.33 |

Table S5. Pairwise comparisons (contrasts) of BMI in COVID-19 pandemic stages. *p*-value adjustment: Benjamini-Hochberg method to control for false discovery rate. Confidence level used: 0.95.

| **Contrast** | **Estimate (**$\boldsymbol{b}$**)** | **Std. estimate (**$\boldsymbol{\beta}$**)** | **SE** | **z-ratio** | **Lower CL** | **Upper CL** | ***p*-value** |
| --- | --- | --- | --- | --- | --- | --- | --- |
| Post_LD I_ - Pre_COVID_ | 0.07 | 0.06 | 0.02 | 2.87 | 0.01 | 0.12 | **< .05** |
| Post_LD II_ - Pre_COVID_ | 0.05 | 0.05 | 0.02 | 2.41 | 0.00 | 0.10 | **< .05** |
| Post_LD II_ - Post_LD I_ | -0.01 | -0.01 | 0.02 | -0.54 | -0.07 | 0.04 | .59 |

Table S6. Estimated marginal trends (emtrends) and confidence intervals of SEB (Slope) in each stage of the COVID-19 pandemic. Confidence level used: 0.95.

| **Time** | **SEB-Trend (Slope)** | **SE** | **Lower CL** | **Upper CL** |
| --- | --- | --- | --- | --- |
| Pre${}_{COVID}$ | -0.13 | 0.01 | -0.16 | -0.10 |
| Post${}_{LD I}$ | -0.14 | 0.02 | -0.18 | -0.10 |
| Post${}_{LD II}$ | -0.17 | 0.02 | -0.20 | -0.14 |

Table S7. Pairwise comparisons of slopes in COVID-19 pandemic stages. *p*-value adjustment: Benjamini-Hochberg method to control for false discovery rate. Confidence level used: 0.95.

| **Contrast** | **Estimate (**$\boldsymbol{b}$**)** | **Std. estimate**  **(**$\boldsymbol{\beta}$**)** | **SE** | **z-ratio** | **Lower CL** | **Upper CL** | ***p*-value** |
| --- | --- | --- | --- | --- | --- | --- | --- |
| Post${}_{LD I}$ - Pre${}_{COVID}$ | -0.01 | -0.01 | 0.02 | -0.61 | -0.06 | 0.03 | .54 |
| Post${}_{LD II}$ - Pre${}_{COVID}$ | -0.05 | -0.04 | 0.02 | -2.61 | -0.09 | -0.00 | **< .05** |
| Post${}_{LD II}$ - Post${}_{LD I}$ | -0.03 | -0.03 | 0.02 | -1.67 | -0.08 | 0.01 | .14 |

**Tables** - Analyses with all months of test

Note: In the analysis with all available test months in the data, adding random slopes for Time by School improved the model fit, which is why it was added to the final model (Table S9). Furthermore, the District dependency in BMIs was smaller (ICC = 0.01) when all test months were included in the data, which is why we did not add random intercepts for District in this case.

Table S8. Results of the type-III sum of squares ANCOVA table with Satterthwaite-approximation.

| **Predictor** | **Sum Sq** | **Mean Sq** | **DF** | **DenDF** | **F-value** | **Pr(>F)** |
| --- | --- | --- | --- | --- | --- | --- |
| Time | 15.90 | 7.95 | 2 | 140.49 | 6.81 | .002 |
| SEB | 288.10 | 288.10 | 1 | 230.24 | 246.88 | **< .001** |
| Gender | 14.51 | 14.51 | 1 | 40934.75 | 12.44 | **< .001** |
| Age | 0.86 | 0.86 | 1 | 40900.81 | 0.74 | .390 |
| Month of Test | 20.70 | 2.59 | 8 | 1116.18 | 2.22 | **< .05** |
| Time x SEB | 11.25 | 5.63 | 2 | 124.65 | 4.82 | **< .01** |

Table S9. Likelihood ratio tests of fixed effects.

| **Model** | **AIC** | **BIC** | **logLik** | **Chisq** | **Param** | ***p*** |
| --- | --- | --- | --- | --- | --- | --- |
| M0 vs. M1 |  |  |  |  |  |  |
| M0 | 125630.0 | 125656.0 | -62812.02 |  | (1\|School) |  |
| M1 | 125607.4 | 125719.7 | -62790.70 | 42.64 | Covariates | **< .001** |
| M1 vs. M2 |  |  |  |  |  |  |
| M1 | 125607.4 | 125719.7 | -62790.70 |  |  |  |
| M2 | 125603.8 | 125733.4 | -62786.91 | 7.6 | Time | **< .05** |
| M2 vs. M3 |  |  |  |  |  |  |
| M2 | 123655.1 | 123784.4 | -61812.55 |  |  |  |
| M3 | 123429.9 | 123567.9 | -61698.94 | 227.22 | SEB | **< .001** |
| M3 vs. M4 |  |  |  |  |  |  |
| M3 | 123429.9 | 123567.9 | -61698.94 |  |  |  |
| M4 | 123423.5 | 123578.7 | -61693.73 | 10.43 | Time x SEB | **< .01** |
| M4 vs. M5 |  |  |  |  |  |  |
| M4 | 123423.5 | 123578.7 | -61693.73 |  |  |  |
| M5 | 123417.5 | 123615.9 | -61685.77 | 15.91 | (Time\|School) | **< .01** |
| Note. Models were successively extended by the following parameters: M0: Unconditional means model with random intercepts for School. M1: Covariates Age, Gender, Month of Test. M2: Predictor Time. M3: Predictor SEB. M4: Predictor Time x SEB interaction. M5: Random slopes for Time. | | | | | | |

Table S10. Fixed and random effect estimates of the fitted (final) linear mixed effects model.

| **Predictor** | **Estimate** | **Std. Beta** | **CI** | **Std. CI** | ***p*** | **Std. *p*** |
| --- | --- | --- | --- | --- | --- | --- |
| Intercept | 0.17 | -0.05 | 0.14 – 0.20 | -0.07 – -0.02 | **<0.001** | **<0.001** |
| Time[LD I] | 0.06 | 0.06 | 0.02 – 0.11 | 0.02 – 0.10 | **0.005** | **0.006** |
| Time[LD II] | 0.06 | 0.05 | 0.02 – 0.10 | 0.01 – 0.08 | **0.003** | **0.007** |
| SEB | -0.13 | -0.15 | -0.15 – -0.12 | -0.17 – -0.13 | **<0.001** | **<0.001** |
| Gender[m] | 0.04 | 0.03 | 0.02 – 0.06 | 0.02 – 0.05 | **<0.001** | **<0.001** |
| Age | 0.00 | 0.00 | -0.01 – 0.02 | -0.01 – 0.01 | 0.390 | 0.390 |
| Jan | -0.02 | -0.02 | -0.06 – 0.02 | -0.06 – 0.02 | 0.276 | 0.276 |
| Feb | -0.02 | -0.01 | -0.07 – 0.04 | -0.06 – 0.03 | 0.562 | 0.562 |
| Mar | 0.05 | 0.05 | 0.00 – 0.10 | 0.00 – 0.09 | **0.033** | **0.033** |
| Apr | -0.04 | -0.04 | -0.17 – 0.08 | -0.15 – 0.07 | 0.479 | 0.479 |
| Aug | -0.06 | -0.06 | -0.14 – 0.01 | -0.13 – 0.01 | 0.103 | 0.103 |
| Sept | -0.01 | -0.01 | -0.05 – 0.03 | -0.05 – 0.03 | 0.611 | 0.611 |
| Oct | 0.05 | 0.04 | 0.00 – 0.09 | 0.00 – 0.08 | **0.037** | **0.037** |
| Nov | 0.02 | 0.02 | -0.02 – 0.06 | -0.01 – 0.05 | 0.260 | 0.260 |
| Time[LD I] x SEB | -0.01 | -0.01 | -0.05 – 0.02 | -0.05 – 0.03 | 0.543 | 0.543 |
| Time[LD II] x SEB | -0.05 | -0.05 | -0.08 – -0.02 | -0.09 – -0.02 | **0.002** | **0.002** |
| Random Effects |  |  |  |  |  |  |
| $\sigma^{2}$ | 1.17 |  |  |  |  |  |
| $\tau_{00school}$ | 0.02 |  |  |  |  |  |
| $\tau_{00school.time0}$ | 0.01 |  |  |  |  |  |
| $\tau_{00school.time1}$ | 0.01 |  |  |  |  |  |
| ICC | 0.02 |  |  |  |  |  |
| N${}_{school}$ | 387 |  |  |  |  |  |
| Observations | 41078 |  |  |  |  |  |
| Marginal/ Conditional R${}^{2}$ | 0.027 / 0.047 |  |  |  |  |  |

Table S11. Estimated marginal means (emmeans) and confidence intervals for BMI in each stage of the COVID-19 pandemic. Confidence level used: 0.95.

| **Time** | **emmean** | **SE** | **Lower CL** | **Upper CL** |
| --- | --- | --- | --- | --- |
| Pre${}_{COVID}$ | 0.17 | 0.01 | 0.14 | 0.20 |
| Post${}_{LD I}$ | 0.23 | 0.02 | 0.19 | 0.28 |
| Post${}_{LD II}$ | 0.22 | 0.02 | 0.18 | 0.26 |

Table S12. Pairwise comparisons (contrasts) of BMI in COVID-19 pandemic stages. *p*-value adjustment: Benjamini-Hochberg method to control for false discovery rate. Confidence level used: 0.95.

| **Contrast** | **Estimate** | **SE** | **z-ratio** | **Lower CL** | **Upper CL** | ***p*-value** |
| --- | --- | --- | --- | --- | --- | --- |
| Post${}_{LD I}$ - Pre${}_{COVID}$ | 0.06 | 0.02 | 2.77 | 0.01 | 0.12 | **< .05** |
| Post${}_{LD II}$ - Pre${}_{COVID}$ | 0.05 | 0.02 | 2.67 | 0.01 | 0.10 | **< .05** |
| Post${}_{LD II}$ - Post${}_{LD I}$ | -0.01 | 0.03 | -0.36 | -0.07 | 0.05 | .72 |

Table S13. Estimated marginal trends (emtrends) and confidence intervals of SEB (Slope) in each stage of the COVID-19 pandemic. Confidence level used: 0.95.

| **Time** | **SEB-Trend (Slope)** | **SE** | **Lower CL** | **Upper CL** |
| --- | --- | --- | --- | --- |
| Pre${}_{COVID}$ | -0.13 | 0.01 | -0.15 | -0.12 |
| Post${}_{LD I}$ | -0.15 | 0.02 | -0.18 | -0.11 |
| Post${}_{LD II}$ | -0.18 | 0.02 | -0.21 | -0.15 |

Table S14. Pairwise comparisons of slopes in COVID-19 pandemic stages. *p*-value adjustment: Benjamini-Hochberg method to control for false discovery rate. Confidence level used: 0.95.

| **Contrast** | **Estimate** | **SE** | **z-ratio** | **Lower CL** | **Upper CL** | ***p*-value** |
| --- | --- | --- | --- | --- | --- | --- |
| Post${}_{LD I}$ - Pre${}_{COVID}$ | -0.01 | 0.02 | -0.61 | -0.05 | 0.03 | .54 |
| Post${}_{LD II}$ - Pre${}_{COVID}$ | -0.05 | 0.02 | -3.10 | -0.09 | -0.01 | **< .01** |
| Post${}_{LD II}$ - Post${}_{LD I}$ | -0.04 | 0.02 | -1.73 | -0.09 | 0.01 | .13 |
